# The Spino-Parabrachial Pathway for Itch

**DOI:** 10.3389/fncir.2022.805831

**Published:** 2022-02-17

**Authors:** Devanshi Piyush Shah, Arnab Barik

**Affiliations:** Centre for Neuroscience, Indian Institute of Science, Bengaluru, India

**Keywords:** PBN, itch, pain, spinal-cord, supra-spinal

## Abstract

Itch-induced scratching is an evolutionarily conserved behavioral response that protects organisms from potential parasites/irritants in their immediate vicinity. How the exposure to a pruritogen is translated to the perception of itch and how that perception drives scratching directed towards the site of exposure remains poorly understood. In this review, we focus on the recent findings that shed light on the neural pathways in the brain that underlie itch-induced scratching. We compare the molecularly defined itch pathways with the known pain circuits as they have anatomical and functional overlap. We review the roles played by the neurons in the spinoparabrachial pathway—comprising of the neurons in the spinal cord and the parabrachial nucleus (PBN), which acts as a hub for transmitting itch information across the brain. Lastly, we deliberate on scratching as a behavioral measure of the intensity of itch and its implication in unraveling the underlying supraspinal mechanisms. In summary, we provide a resource on the recent advances and discuss a path forward on our understanding of the neural circuits for itch.

## Introduction

Nocifensive behaviors are critical for survival—they act as the first line of defense against potentially harmful stimuli present in our immediate environment. One such nocifensive behavior is scratching, which is uniquely induced by itch. When a mosquito bites, histamine is released at the site of injury by the immune cells stimulating the histamine receptor-expressing free nerve endings of the primary sensory neurons present in the superficial layers of the skin. The sensory neurons activate projection neurons in the dorsal horn of the spinal cord, which forwards the information to the brain, giving rise to the perception of itch and driving the motor reflex to scratch. The scratching response is targeted to the skin area where the nerves are stimulated and continues until the sensation of itch subsides. The scratching behavior ensures our attention to the site of itch, provides relief, and makes us aware of the presence of vector-borne disease-causing insects or allergens. How scratching is initiated, how it is targeted to the location of the itch, how the scratching subsides with the cessation of itch, and how scratching an itch provides a sense of relief remains poorly understood. Recent advances in the techniques to genetically target, anatomically map, monitor, and manipulate the activity of a select group of neurons (Lerner et al., 2016; Luo et al., 2018; Wolff and Ölveczky, 2018) have revealed specific neural circuits in the spinal cord and the brain that process itch information. Moreover, scratching inhibits itch by causing pain; circuits for pain are known to transmit itch, and thus pain circuits can form the basis to further our insights on itch circuitries (Ikoma et al., 2003; Schmelz, 2010). In this short review, we discuss: (a) recent findings on the overlapping and distinct brain circuits that drive and mediate responses to pain and itch, (b) the role of PBN as a critical node through which itch information is distributed across the central nervous system, and (c) the potential of scratching as a behavioral measure in studies designed to decipher the circuit mechanisms underlying itch.

## Interactions Between Pain and Itch in The Spinal Projection Neurons

The perceptions of pain and itch are distinct and characterized by specialized protective behaviors such as withdrawal and escape in response to pain and scratching to itch. Previously, itch was thought to be a form of mild pain (Handwerker, 2014); however, recent evidence indicates that itch and pain are perceived and processed by independent circuits in the nervous system. Notably, pain and itch share an antagonistic relationship. For example, scratching causes pain and inhibits itch, while administration of analgesic opioids results in intense itch (Davidson and Giesler, 2010). Since the central circuit mechanisms for pain are relatively well-delineated, determining how pain circuits respond and modulate itch can provide critical insights into the central mechanisms of itch.

Itch (Hoon, 2015; Chen and Sun, 2020) and pain (Basbaum et al., 2009; Dubin and Patapoutian, 2010) from the skin are transmitted by dedicated sensory receptors from the skin to the dorsal horn of the spinal cord. Both the pain and itch information impinges upon local interneurons and projection neurons in the dorsal horn. Recent evidence points to the existence of pain- and itch-specific interneurons. Inhibition and ablation of a molecularly defined population of interneurons abolish itch but not pain (Liu et al., 2019). Similarly, mechanically evoked itch is gated by an independent group of peptidergic interneurons without any bearing on pain (Bourane et al., 2015). However, it remains unclear if there is a group of spinal projection neurons that are dedicated to carrying pruritic information to the brain. The genes encoding gastrin gene-related peptide (GRP) and GRP receptor (GRPR) are expressed in two distinct populations of synaptically connected neurons in the dorsal horn of the spinal cord and this local circuitry is critical for both itch transmission from the periphery to the brain and itch-induced scratching (Bautista et al., 2014; Bardoni et al., 2019; Chen and Sun, 2020). Recently, it was shown that the GRPR-expressing interneurons synapse onto the Tacr1 projection neurons—a projection neuron population known to carry noxious information from the dorsal horn to the PBN and the thalamus (Todd, 2010). Retrograde and co-labeling studies indicate that the Tacr1 and GRPR populations do not overlap, and the GRPR neurons do not project to the brain (Bardoni et al., 2018). Chemogenetic and optogenetic stimulation of the GRPR neurons induces spontaneous itch behaviors, and the itch can be suppressed by local administration of chemical Tacr1 inhibitor (Bardoni et al., 2018). Thus, the evidence suggests that the GRP-GRPR local spinal circuit might signal itch to the brain *via* the Tacr1 projection neurons. Though, a small population of spinal GRPR neurons were found to project to the brain and thus, can potentially be involved in the central transmission of itch independent of the Tacr1 projection neurons (Mu et al., 2017). Spinal ablation of Tac1 (gene encoding Substance P, peptide ligand for Tacr1) expressing neurons, which significantly overlap with Tacr1 (Barik et al., 2021), reduced non-histaminergic itch (Huang et al., 2019). This finding is in agreement with the pharmacological blockade and ablation studies that indicate that the Tac1 and Tacr1 neurons, respectively, are required for acute and chronic itch (Carstens et al., 2010; Akiyama et al., 2015). However, chemogenetic and optogenetic stimulation of Tacr1 spinal projection neurons (Deng et al., 2020; Barik et al., 2021) drive spontaneous pain-induced protective behaviors and suppress itch (Barik et al., 2021). In an independent study, chemogenetic activation of Tacr1 neurons promoted itch and lacked spontaneous pain behaviors (Sheahan et al., 2020). This contradiction can be explained due to the usage of different genetic strategies to target the Tacr1 population. In disagreement with previous findings, this study also indicates that the Tacr1 neurons are primarily interneurons and they partially overlap with GRPR neurons (Sheahan et al., 2020). Recent studies have revealed the existence of other distinct spinal projection neurons apart from ones expressing Tacr1. Gpr83 expressing projection neurons do not express Tacr1 and are specifically tuned to mechanical stimuli (Choi et al., 2020), and thus might mediate non-histaminergic mechanical itch. Another recent study explicitly designed to uncover the molecular identity of dorsal horn projection neurons that are tuned to pain and itch stimuli found that retrogradely labeled neurons from the brain expressing genes such as Cck, Nptx2, Nmb, and Crh are activated by itch (Wercberger et al., 2021). However, Cck and Nptx2 are a subset of Tacr1 projection neurons (Wercberger et al., 2021). Taken together, the existence of itch-specific spinal projection neurons still remains unclear. The well-studied projection neuron population expressing Tacr1 seem to receive pruritic inputs but are tuned to and preferentially involved in pain-induced behaviors. The transmission of itch at the spinal projection neuron-level might follow a population coding model where labeled/dedicated neurons for somatosensory modalities interact to facilitate itch perception in the brain (Pouget et al., 2000; Ma, 2010).

## Pbn as A Gateway to Understanding Itch Processing in The Brain

Macroscopic imaging techniques such as functional Magnetic Resonance Imaging (fMRI) and Positron Emission Tomography (PET) scans in human subjects reveal the involvement of a range of brain regions such as the thalamus, primary and secondary somatosensory cortex (S1 and S2), insular cortex (IC), anterior cingulate cortex (ACC), central amygdala (CeA), motor cortex, basal ganglia, putamen, PBN, and periaqueductal gray (PAG) (Davidson and Giesler, 2010; Akiyama and Carstens, 2013; Mochizuki et al., 2019) in itch. In contrast, mechanistic studies in animal models to elucidate cells and circuits underlying itch and scratching have only intensified recently. Simultaneous antidromic stimulation in the thalamus and extracellular recordings in the primate lumbar spinal cord facilitated the identification of itch-responsive neurons in the ventral posterolateral/inferior nuclei and the supra/medial geniculate nuclei (Davidson et al., 2007, 2009, 2012). Lateral PBN neurons, on which the pruritic information from the spinal cord converge, are necessary for both histaminergic and non-histaminergic itch (Mu et al., 2017). PAG stimulation is sufficient to generate spontaneous scratching in the absence of stimuli (Gao et al., 2019; Samineni et al., 2019). The central nucleus of the amygdala (CeA), which receives excitatory inputs from the PBN, and in turn, has inhibitory projections to the PAG and PBN, contains neurons that are sufficient to generate itch and are involved in itch-associated aversion (Chen et al., 2016; Sanders et al., 2019; Samineni et al., 2021). Itch-induced scratching increases dopaminergic neuronal activity in the VTA, and this activity is required for gaining pleasure from scratching (Yuan et al., 2018; Su et al., 2019). Experiments done with the anterograde transsynaptic HSV viral particles to find putative itch perceptive neurons using GRP interneurons in the spinal cord as a starting point have indicated the involvement of the ventrolateral PAG, dorsomedial hypothalamus (DMH), rostral ventromedial medulla (RVM), PBN, central amygdala (CeA), thalamus, S1, and S2 (Albisetti et al., 2019). Taken together, data from studies done in human subjects and animal models indicate that several brain nuclei are likely to be instrumental in itch perception and the resultant behavior. However, it remains unclear how these disparate brain regions together give rise to the itch percept and allow animals to appropriately respond to it.

**Figure 1:**
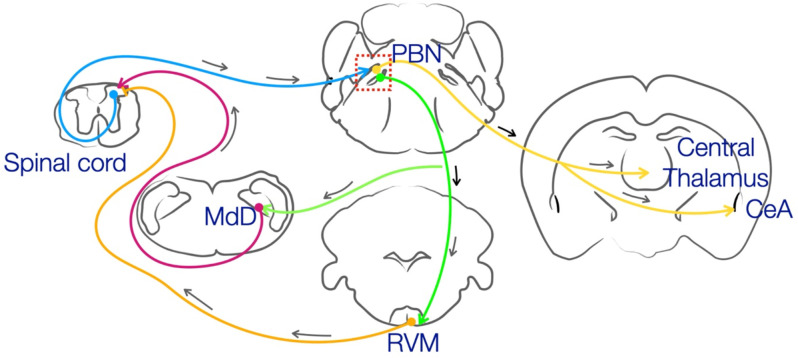
Spinal projection neurons carrying somatosensory information originate from the superficial layers of the dorsal horn and project to the PBN. The information from the PBN is relayed to the mid- and fore-brain regions such as the central thalamus and the CeA to mediate the aversive and emotional effects of pain and itch. Through the projections to the RVM and MdD and their direct connections with the spinal cord neurons, PBN can modulate the threshold for both pain and itch. PBN, parabrachial nucleus; RVM, rostral ventromedial medulla; CeA, central amygdala; MdD, Dorsal Medullary Reticular Formation.

PBN is primarily constituted of excitatory projection neurons (Krukoff et al., 1993; Barik et al., 2018, 2021; Huang et al., 2021) and a small population of inhibitory interneurons (Chiang et al., 2020). Spinal inputs to the PBN from a diverse set of functionally distinct projection neurons expressing Tac1, Tacr1, and Grp83 which carry somatosensory information from the spinal cord (Huang et al., 2019; Choi et al., 2020; Deng et al., 2020), terminate at distinct coordinates of the lateral PBN. Axon terminals of lateral PBN neurons that receive direct spinal inputs project to the CeA, IC, thalamus, hypothalamus, PAG, reticular formation, RVM, and VTA (Fulwiler and Saper, 1984; Gauriau and Bernard, 2002; Barik et al., 2018; Huang et al., 2021). Thus, PBN has access to the cortical sensory areas *via* the thalamus, the limbic system *via* the amygdala, the autonomic nervous system through the hypothalamus, pain modulatory system *via* the RVM, and MdD (Figure 1). The autonomic and arousal components of the itch pathway can be potentially mediated by the bi-directional communication between the PBN and the insular cortex (Grady et al., 2020). Through its direct projections to the VTA, PBN can modulate scratch-induced reward and itch-induced aversion (Coizet et al., 2010; Yuan et al., 2018; Su et al., 2019; Yang et al., 2021). The activation of PBN neurons corresponds to bouts of scratching, implying that the motor output of scratching can be driven or mediated by PBN neurons. PBN neurons may also influence the quantum of itch by driving stress through inputs to the paraventricular thalamus (PVT). PBN forms closed-loop circuits with spinal cord dorsal horn *via* the MdD and can be instrumental in driving motor outputs in response to high-threshold somatosensory stimuli (Barik et al., 2018). Recently, it has been shown that RVM ON cells, which are the spinally projecting pro-nociceptive neurons (Watkins et al., 1980; Fields et al., 1995), are sufficient to inhibit itch when stimulated (Follansbee et al., 2021). RVM ON cells receive modulatory inputs from pro-nociceptive structures such as the PAG, PBN, and MdD (*via* PAG) (Beitz et al., 1983; Leite-Almeida et al., 2006; Roeder et al., 2016; Chen et al., 2017; Chiang et al., 2019) and thus, these inputs to the RVM may explain how pain inhibits itch in the brain. Taken together, owing to the diverse somatosensory inputs and post-synaptic connections across the brain, PBN may mediate and modulate the behavioral and physiological responses to itch.

## Scratching as A Measure of Itch and A Tool to Dissect Brain Pathways

In studies designed to shed light on the brain circuits involved in itch, it is crucial to objectively ascertain its severity in human subjects and preclinical animal models. Traditionally, human studies have involved detailed questionnaires to self-report itch severity on a predefined numerical scale or selection of listed words to indicate the vigor of the itch sensation (Dawn et al., 2009). However, self-reporting of itch severity can be subjective and, more importantly, cannot account for itching while the subject is sleeping or engaged in an activity that draws their attention away from the itch. Thus, there is a need for developing robust ways to measure the itch severity in human subjects (Wimalasena et al., 2021) in an unbiased manner. In the common animal models for itch—mice and rats, itch severity is measured by the number of scratches in a given time. The same measure for itch can be applied to human subjects to determine the urgency, frequency, and intensity. Recent technological innovations in ultrasensitive miniaturized sensors that can report the acceleration and position of an object in 3D space in real-time can be taken advantage of to report scratching objectively both in humans and animals. Deep learning-based pose estimation tools such as DeepLabCut and DAANCE (Mathis et al., 2018; Dunn et al., 2021) can be exploited to gain a deeper understanding of itch-induced scratching by high-resolution 3D tracking of body parts and automated behavioral classification. The advantage of the camera-based analysis of scratching is that the tools can be used across species since it will not depend on the physical size of sensors that are required to vary between mice, rats, or primates. Notably, this strategy of using scratching as the primary measure for the intensity of itch in human subjects and animal models can enhance the impact of the findings from basic behavioral studies (Wimalasena et al., 2021).

The motor response of scratching has provided us with insights into the workings of the CNS for more than a century (Sherrington, 1906). Itch-induced scratching is dynamic and unlike the reflexive response to pain such as withdrawal and escape, scratching sets in tens of seconds or minutes after exposure to pruritogens. Scratches occur in bouts, which entails rapid successive movement of limbs targeting sharp edges of nails to the skin area from where the itch has originated. Each bout is terminated when a temporary relief from itch is perceived; however, scratching continues until the feeling of itch is completely gone. The bouts tend to occur in higher frequencies right after the pruritogen exposure, and the frequency is received with time as greater relief is achieved. In rodents, each scratching bout ends with a paw lick. Thus, the motor behavior of scratching is intricately linked with the sensation of itch and the achievement of relief. How, in the brain, the sensation of itch, the cessation of the itch by the pain caused by scratching, and the comfort provided by scratching interact at the level of cells and circuits remains unknown. The spinal projection neurons, the sole conduit for transmitting both itch and pain information to the brain, might be a key to deciphering the underlying circuitry for scratch-induced itch relief.

In the studies to reveal the brain structures, circuits, and cells involved in itch, two types of itch stimuli have been used: histaminergic and non-histaminergic pruritic stimuli (LaMotte et al., 2014). Non-histaminergic itch is of greater clinical concern than histaminergic itch since histamine release is not the causal agent for most chronic itch conditions. In addition, the histaminergic itch can be resolved with available antihistamines. Among pruritic agents used to model non-histaminergic itch, most prominent are: spicules of cowhage (*Mucuna pruriens*; Papoiu et al., 2011), which are applied on the skin to mimic mechanical itch in both laboratory animals and humans; electrical stimulation, which activates unmyelinated fibers in the skin; chemical irritants such as chloroquine which are injected subdermally in mice/rats to elicit transient itch. How histaminergic and non-histaminergic itch are differentially encoded in the brain remains to be investigated. It will be important to know if the molecularly defined and anatomically circumscribed neuronal populations specifically encode non-histaminergic itch. Notably, the frequency of scratching might vary depending on the nature of the pruritogen. A detailed study of scratching induced by histaminergic and non-histaminergic pruritogens may shed light on the underlying neural circuits.

## Future Directions

Studies geared towards unraveling the central mechanisms of itch have shed light on several genetically and anatomically defined circuits over the last decade. Especially from the research done in preclinical models such as mice and rats, it is apparent that the ascending pathways from the spinal cord hold the key to a better understanding of circuits for itch. To that end, in this minireview, we have: (a) discussed our current state of understanding of the spinal projection neuron population in the dorsal horn of the spinal cord; (b) explored how the PBN can facilitate understanding of the brain mechanisms for itch due to its wide-ranging downstream neural networks; and (c) expanded on the rationale to focus on the scratching behavior as a primary measure in delineating the central circuits for itch.

Dorsal projection neurons synapse onto brain areas that remain unexplored in the context of itch. For example, Tacr1 projection neurons synapse onto gracile and cuneate nuclei in the brainstem, which receive direct light threshold mechanoreceptor and proprioceptor inputs, respectively. Thus, the gracile nucleus can potentially be instrumental in integrating light threshold and pruritic information and communicating to the sensory cortex *via* its primary target, the thalamus. MdD, with its reciprocal connections to the dorsal horn spinal neurons, is well known to mediate pain; however, how this circuitry may intervene in itch remains to be explored. Tacr1 projection neurons also have direct access to the central thalamus which allows them to drive arousal, which in turn can modulate itch. Further, recently developed strategies to define central nervous system-wide networks of downstream neurons with artificial activation of target neuron population and brain-wide analysis of immediate early gene (IEG) expression (Renier et al., 2014, 2016; Barik et al., 2018) for pruriceptive spinal projection neurons can uncover novel itch circuits.

PBN acts as a hub for relaying various aversive stimuli, including pain and itch (Chiang et al., 2019). One of the intriguing questions in circuit neuroscience that several groups actively pursue is how the brain state dictated by physiological necessities such as sleep, hunger, taste, and thirst affect pain and itch, and vice-versa (Palmiter, 2018; Kang et al., 2020). PBN is central to this interaction between somatosensory modalities such as pain and itch and physiological brain states (Alhadeff et al., 2018, 2020; Phua et al., 2021). However, it remains unclear how the somatosensory systems and brain states compete in the PBN and whether PBN neurons prioritize behavior depending on these competitive interactions. PBN is rich in neuropeptides, and it is not known what roles do they play in competitive arrangements between sensory and physiological stimuli. Experimental paradigms involving single-cell physiology, gene expression, *in vivo* imaging, and novel behavioral assays will be instrumental in answering these open questions. Notably, a detailed analysis of itch-induced scratching will be paramount in realizing the goals mentioned earlier. Further, we propose that the spino-parabrachial pathway, constituting the spinal projection neurons that terminate in the PBN and the PBN neurons that transmit itch information across the brain, might be the key to understanding the “itch matrix” akin to the “pain matrix” in the brain (Bushnell et al., 2013). The “itch matrix” is comprised of the brain regions (Najafi et al., 2021) whose activity and interactions are necessary and sufficient for generating the itch perception, the behavioral output of scratch, and their resultant physiological consequences.

## Author Contributions

DS and AB conceptualized researched literature and wrote the manuscript. All authors contributed to the article and approved the submitted version.

## Conflict of Interest

The authors declare that the research was conducted in the absence of any commercial or financial relationships that could be construed as a potential conflict of interest.

## Publisher’s Note

All claims expressed in this article are solely those of the authors and do not necessarily represent those of their affiliated organizations, or those of the publisher, the editors and the reviewers. Any product that may be evaluated in this article, or claim that may be made by its manufacturer, is not guaranteed or endorsed by the publisher.
